# Effects of intravenous iron on fibroblast growth factor 23 (FGF23) in haemodialysis patients: a randomized controlled trial

**DOI:** 10.1186/s12882-016-0391-7

**Published:** 2016-11-16

**Authors:** Matthew A. Roberts, Louis Huang, Darren Lee, Robert MacGinley, Stefanie M. A. Troster, Annette B. Kent, Sukhvinder S. Bansal, Iain C. Macdougall, Lawrence P. McMahon

**Affiliations:** 1 Eastern Health Clinical School, Monash University, Box Hill, VIC Australia; 2 Department of Renal Medicine, Eastern Health, Box Hill, VIC Australia; 3 Institute of Pharmaceutical Science, King’s College, London, UK; 4 Department of Renal Medicine, King’s College Hospital, London, UK

**Keywords:** Fibroblast growth factor-23, Hepcidin, Haemodialysis, Iron infusion, Randomized controlled trial

## Abstract

**Background:**

Intravenous iron affects serum levels of intact fibroblast growth factor-23 (iFGF23) and its cleavage product c-terminal FGF23 (cFGF23) in iron-deficient people with normal renal function. We hypothesized that intravenous iron modulates iFGF23 and cFGF23 in haemodialysis patients differently according to the type of iron used.

**Methods:**

Prevalent, stable haemodialysis patients requiring protocol-based intravenous iron therapy were randomized to a single 200 mg dose of either ferric carboxymaltose (FCM) or iron sucrose (IS). The primary outcome was change in iFGF23 and cFGF23 from pre-infusion to Day 2 post-infusion. Serum hepcidin, ferritin and phosphate were also measured. Pair-wise comparisons utilised the Wilcoxon rank sum test; linear mixed models with an interaction term for treatment and time evaluated between-group effects.

**Results:**

Forty-two participants completed the study. In those randomized to FCM (*n* = 22), median (interquartile range) values pre-infusion and Day 2, respectively, were 843 pg/mL (313–1922) and 576 pg/mL (356–1296, *p* = 0.05) for iFGF23, 704RU/mL (475–1204) and 813RU/mL (267–1156, *p* = 0.04) for cFGF23, and 1.53 mmol/L (1.14–1.71) and 1.37 (1.05–1.67, *p* = 0.03) for phosphate. These parameters did not change following IS. Both serum ferritin (*p* < 0.001) and hepcidin (*p* < 0.001) increased in both groups, and the increase in hepcidin was greater in the FCM group (*p* = 0.03 for between-group difference).

**Conclusions:**

Contrary to iron-deficient people with normal renal function, haemodialysis patients given protocol-driven intravenous FCM demonstrated a fall in iFGF23 and a rise in cFGF23, changes not evident with IS. This suggests a differential effect of intravenous iron treatment according to both formulation and renal function.

**Trial registration:**

Australian and New Zealand Clinical Trials Register ACTRN12614000548639. Registered 22 May 2014 (retrospectively registered).

**Electronic supplementary material:**

The online version of this article (doi:10.1186/s12882-016-0391-7) contains supplementary material, which is available to authorized users.

## Background

Serum concentrations of fibroblast growth factor 23 (FGF23) rise early in the course of chronic kidney disease (CKD) [1] and are strongly correlated with adverse clinical outcomes [2, 3]. However, whether this association is causally related or simply reflective of concurrent pathophysiological processes is not known, for although a modulatory role for FGF23 in phosphate homeostasis is well recognized, substantive control over serum levels is also exerted through intracellular proteolysis, and other key modulatory factors are still being elucidated [4]. Specifically, recent publications suggest a regulatory effect between FGF23 and iron homeostasis, potentially mediated via hepcidin and hypoxia inducible factor (HIF) [5, 6]. A recent series of in vivo and in vitro experiments demonstrated that the effects of inflammation and iron deficiency on FGF23 is mediated through HIF-1α; and induction of functional iron deficiency by administration of exogenous hepcidin increased osseous FGF23 mRNA expression and serum levels of inactive c-terminal FGF23 (cFGF23), whereas concentrations of the intact molecule (iFGF23) were unaffected [7].

Intravenous iron is widely and extensively used to manage iron deficiency anaemia in people without and with CKD [8, 9], and effects on circulating FGF23 concentrations have proven variable. Increased levels of iFGF23 (with post-infusion hypophosphatemia) have been demonstrated in people without CKD in case reports [10, 11] as well as one prospective study [12]; and a randomized controlled trial of iron-deficient women showed an increase in iFGF23 but dramatically reduced cFGF23 following intravenous ferric carboxymaltose [13]. Administration of intravenous iron dextran [14] or saccharated ferric oxide [15] increased iFGF23 in separate studies of haemodialysis (HD) patients, without cFGF23 being measured.

No randomised studies of changes in FGF23 following intravenous iron have been conducted in HD patients. We therefore aimed to determine whether treatment of HD patients with two commonly used formulations at standard doses affected iFGF23 and cFGF23 differently.

## Methods

The D-IDENTIFY Study compared two formulations of intravenous iron therapy, ferric carboxymaltose (FCM) and iron sucrose (IS), in stable HD patients. The study was approved by the Eastern Health Human Research Ethics Committee (E13-1314) and study conduct adhered to the Declaration of Helsinki.

### Participants

Prevalent patients were recruited from HD units attached to a single centre (Eastern Health) if they were (1) receiving thrice-weekly HD for 3 months or more at the time of enrolment, (2) aged 18–90 years, and (3) required intravenous iron therapy based on protocol guidelines. Exclusion criteria were (1) inability to give written informed consent, (2) clinical evidence of active infection, (3) active malignancy, (4) receipt of a blood transfusion within the preceding 4 weeks, (5) severe anemia defined as hemoglobin <85 g/L, (6) previous hypersensitivity to intravenous IS or FCM, and (7) recent (within last 4 weeks) introduction or change in dose of erythropoiesis stimulating agent (ESA), phosphate binders, vitamin D analogues, or cinacalcet. Furthermore, no participant received iron in the preceding 6 weeks before recruitment. If patients had received iron at time of screening, they could be re-screened 6 weeks after the last dose.

The national guidelines recommend intravenous iron for anemic patients receiving an ESA when serum ferritin is <200 μg/L or iron saturation <20%, and for those not receiving an ESA when serum ferritin is <100 μg/L or iron saturation <20% [16]. At our institution, iron studies are measured every 2 months and patients receive five weekly doses of 100 mg of iron when the above criteria are met. Patients can receive iron if they fall outside of the ferritin and iron saturation ranges if the clinician feels there are other reasons why iron might be indicated.

### Procedures

After obtaining written informed consent and confirming that participants met the criteria for intravenous iron, participants were randomized to either intravenous FCM (Ferinject, Vifor Pharma Australia Pty Ltd) 200 mg or IS (Venofer, Aspen Pharmacare Australia Pty Ltd) 200 mg, each administered as a single dose. We chose to give a higher single dose than the usual 100 mg (weekly) dose because participants could not receive further intravenous iron until end of study (42 days). A dose of 500 mg or above, as is given to people not requiring dialysis, were considered too far from clinical practice in HD patients.

Randomization was performed using a permuted block design with block sizes of 2, 4, 6 and 8, and allocation was within blocks in a 1:1 ratio using the ‘random allocation’ function of Stata version 11.2 (College Station, Texas). Investigators and participants were not blinded to treatment allocation but both were unaware of test results during the study.

After randomization, clinical and demographic data were collected and blood was taken on Days −14, −7, as well as immediately pre-infusion, and Days 2, 7, 21 and 42 post-infusion. Samples were collected from the arterial needle into serum and EDTA tubes before HD, left to stand and clot (serum), centrifuged within 3 h, and then aliquots of serum and plasma were stored at −80 °C for subsequent analysis.

### Study endpoint

The primary endpoint was the change in iFGF23 and cFGF23 from Baseline to Day 2 after administration of intravenous iron therapy. Secondary endpoints included the change in serum hepcidin, ferritin and phosphate.

### Sample analysis

Both iFGF23 and cFGF23 were measured in duplicate by enzyme-linked immunosorbent assays (ELISA) with a human FGF23 immunometric assay, and reported as pg/mL (Kainos, Shizuoka, Japan), and RU/mL (Immutopics, San Clemente, USA), respectively. In our laboratory, the intra-plate co-efficient of variation (CV) using duplicates from the patient cohort was 5.8% for iFGF23 and 5.7% for cFGF23. These two FGF23 assays do not have clinically significant pre-analytic stability issues [17].

Serum hepcidin concentrations were measured by tandem mass spectrometry using a published method [18] which was updated using a Waters ACQUITY Ultra-Performance Liquid Chromatography system with a Xevo TQ-S mass spectrometer [19] (described in detail in this reference’s online supplement). All other laboratory analyses were performed by the clinical laboratory service at Eastern Health, a National Association of Testing Authorities accredited service.

### Statistical analysis

From a previous randomized controlled trial [13], it was considered a sample size of 20 participants in each group would provide 90% power to detect a within-group and between-group change of 0.8 times the baseline standard deviation of FGF23 concentrations.

Measurements at Study Days −14, −7 and 0 were used to estimate pre-infusion week-to-week variation in iFGF23. The within-subject CV was derived from the variance of the residuals of the multilevel mixed-effects linear regression with random effects at the participant level. Because of this result, we used the mean of the three pre-infusion iFGF23 measures as the baseline value for iFGF23. For the other variables, the single serum sample taken immediately prior to the iron infusion was used.

We created linear mixed models to assess the change in measured variable from baseline to following iron infusion. As fixed effects, we included iron formulation (FCM versus IS) and time, and for random effects we included a random intercept for participants. To determine whether the change in measured variable following the iron infusion differed by iron formulation, an interaction term for treatment and time was introduced. As the models included observations at each time point, the *p* value for interaction for the change at Day 2 is reported. Model assumptions were tested by inspection of plots of residuals to look for deviations from normality.

For baseline comparisons, data is presented as mean ± standard deviation or median (interquartile range) according to the distribution of the variable and appropriate parametric or non-parametric tests applied. Pair-wise comparison of Day 0 and Day 2 values of measured parameters was performed using the Wilcoxon signed-ranks tests, as variables were not normally distributed. Comparison of measures at all time points with baseline adjusted for multiple comparisons was performed using the Kruskal-Wallis test. Analyses were performed using Stata version 11.2 (Satacorp, College Station, Texas).

## Results

**Fig. 1:**
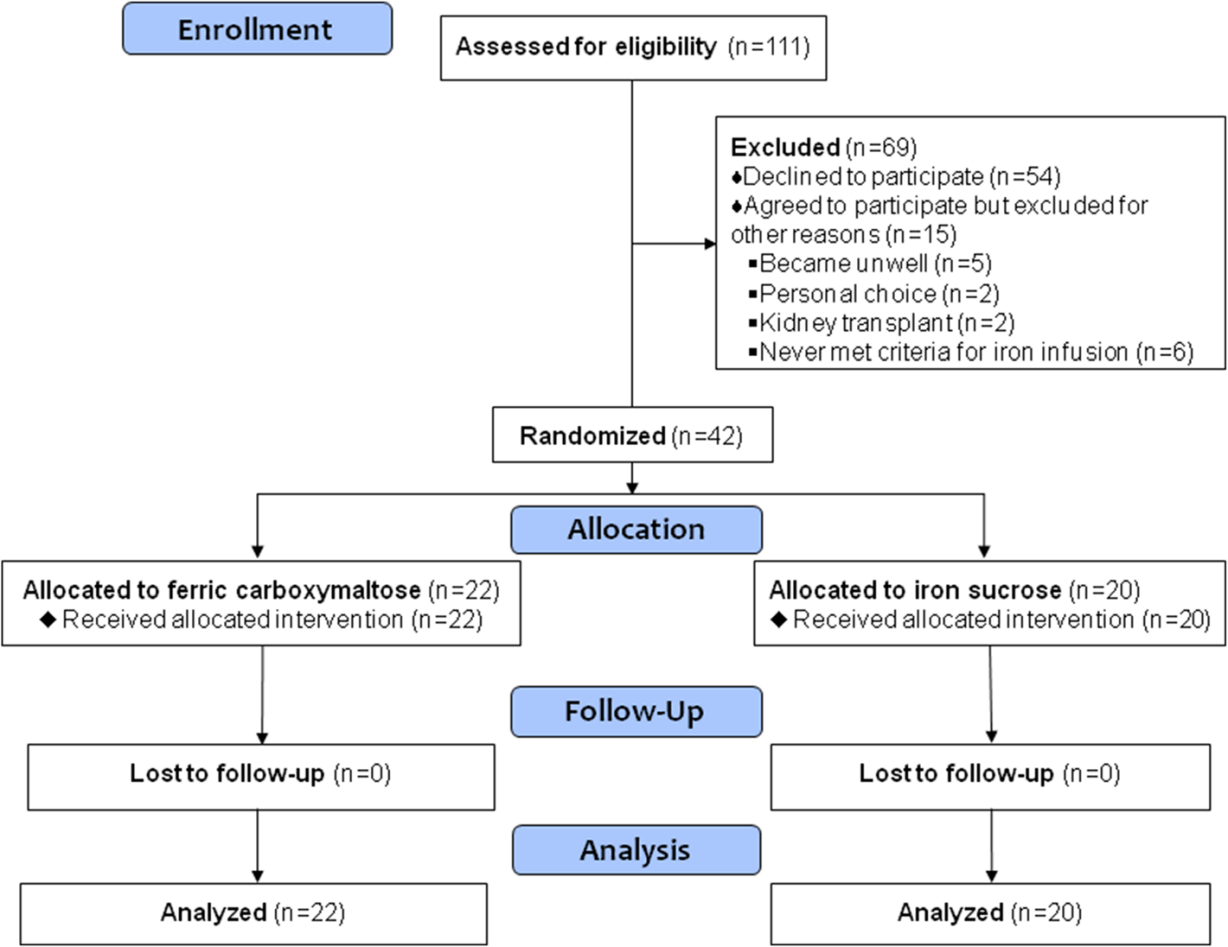
CONSORT Flow diagram of patient enrolment

Of 111 patients approached for participation, 54 declined to participate and 15 met exclusion criteria (Fig. 1) leaving 42 randomized to receive FCM (FCM Group, *n* = 22) or IS (IS Group, *n* = 20). The baseline clinical characteristics (Table 1) in each group were similar. Baseline laboratory values (Table 2) were mostly similar but iFGF23 was substantially higher at baseline in the FCM Group, although the difference was not statistically significant. Participants were reasonably iron replete according to mean ferritin and transferrin saturation values and 14 participants had either ferritin or iron saturation above the guideline targets when prescribed their iron (8 in the FCM Group and 6 in the IS Group).

**Table 1:** Baseline characteristics of participants by treatment allocation to ferric carboxymaltose or iron sucrose

	Ferric carboxymaltose (*n* = 22)	Iron sucrose (*n* = 20)	*P*
Age	70.9 (66.7–76.8)	75.1 (56.1–79.8)	0.58
Male sex	16 (73%)	14 (70%)	0.85
Diabetes	6 (27%)	9 (45%)	0.23
Cardiovascular disease	14 (64%)	15 (75%)	0.43
History of parathyroidectomy	1 (5%)	1 (5%)	0.95
Cause of ESKD			0.82
Diabetes	5 (23%)	6 (30%)	
Glomerulonephritis	2 (9%)	2 (10%)	
Vascular	3 (14%)	4 (20%)	
Other	12 (55%)	8 (40%)	
Haemodialysis (hr per week)	13.5 ± 1.5	13.2 ± 1.6	0.52
URR (%)	77.6 ± 5.8	75.3 ± 6.4	0.23
Dialysis vintage (months)	44.3 (21.7–76.6)	34.6 (18.9–64.6)	0.35
Dry weight (kg)	76.9 ± 15.8	76.8 ± 15.0	0.99
Urine volume ≥500 mL/day	7 (32%)	9 (45%)	0.38
BMI (kg/m^2^)	26.5 ± 4.2	27.8 ± 5.4	0.41
SBP (post-dialysis)	130.0 ± 20.0	130.8 ± 24.9	0.91
DBP (post-dialysis)	64.0 ± 14.2	59.0 ± 16.0	0.29
Medications			
ESA (epoetin-β equivalent, U/week)	3800 (0–5000)	2000 (0–5800)	0.62
Receiving any ESA	16 (73%)	11 (55%)	0.23
1,25(OH_2_) Vit D	12 (55%)	8 (40%)	0.35
25(OH) Vit D	9 (41%)	6 (30%)	0.46
Cinacalcet	12 (55%)	7 (35%)	0.20
Calcium carbonate (500 mg/ 600 mg)	13 (59%)	9 (45%)	0.36
Lanthanum	11 (50%)	9 (45%)	0.75
Sevelamer	7 (32%)	8 (40%)	0.58
Multiple binders	10 (45%)	6 (30%)	0.30

*URR* urea reduction ratio, *BMI* body mass index, *SBP* systolic blood pressure, *DBP* diastolic blood pressure, *ESA* erythropoiesis stimulating agent

**Table 2:** Laboratory variables of participants by treatment allocation to ferric carboxymaltose or iron sucrose

	Ferric carboxymaltose *n* = 22	Iron sucrose *n* = 20	*P*
Hemoglobin (g/L)	111 ± 9	115 ± 10	0.14
Iron (μmol/L)	14.0 (11.0–16.0)	10.5 (8.5–17.5)	0.31
Ferritin (μg/L)	211 ± 133	187 ± 130	0.55
Transferrin Saturation (%)	29 ± 9	27 ± 12	0.64
Transferrin (g/L)	2.2 ± 0.4	2.2 ± 0.5	0.65
Hepcidin (ng/mL)	7.8 (2.7–12.6)	3.4 (2.7–8.8)	0.24
iFGF23 (pg/mL)	843 (313–1922)	381 (245–1526)	0.33
cFGF23 (RU/mL)	704 (475–1204)	710 (448–1548)	0.76
PTH (pmol/L)	40.0 (20.6–68.3)	31.2 (16.6–42.2)	0.22
Ca (corrected) (mmol/L)	2.30 ± 0.17	2.31 ± 0.15	0.90
PO_4_ (mmol/L)	1.53 ± 0.44	1.42 ± 0.44	0.41
Albumin (g/L)	35.5 ± 2.5	34.6 ± 2.8	0.27
CRP (mg/L)	4.5 (2.0–6.0)	3.0 (2.0–5.0)	0.33

*RU/mL* relative units per mL, *CRP* C-reactive protein

### Variability of iFGF23

Based on three weekly pre-infusion measures, the within-subject CV for iFGF23 was 25%, with 96% between-subject variability.

### Change from baseline to day 2 in randomized groups

**Fig. 2:**
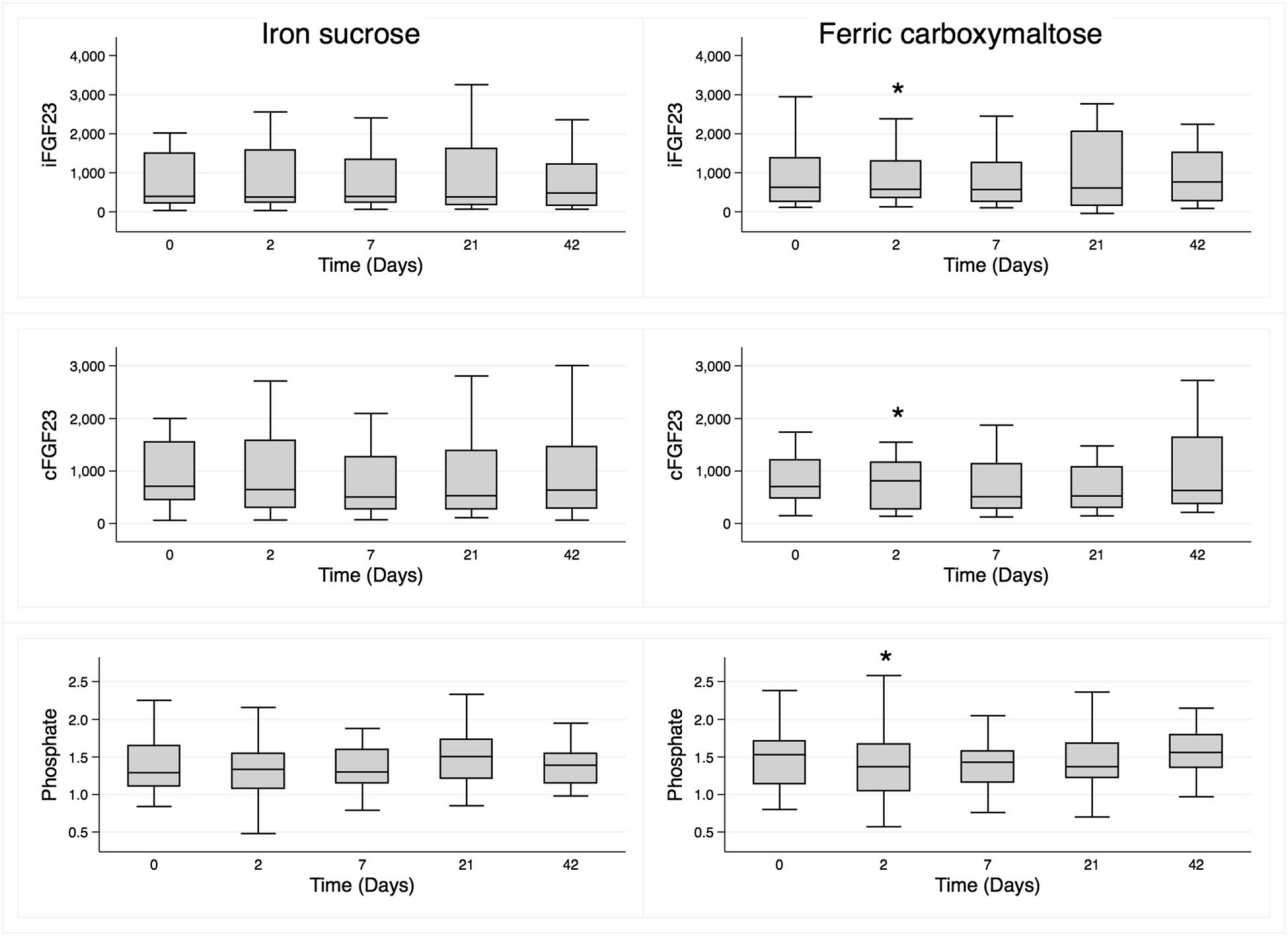
Median (interquartile range) levels over the course of the study of iFGF23 (pg/mL), cFGF23 (RU/mL) and phosphate (mmol/L) in participants treated with iron sucrose (*left panels*) and ferric carboxymaltose (*right panels*)

**Fig. 3:**
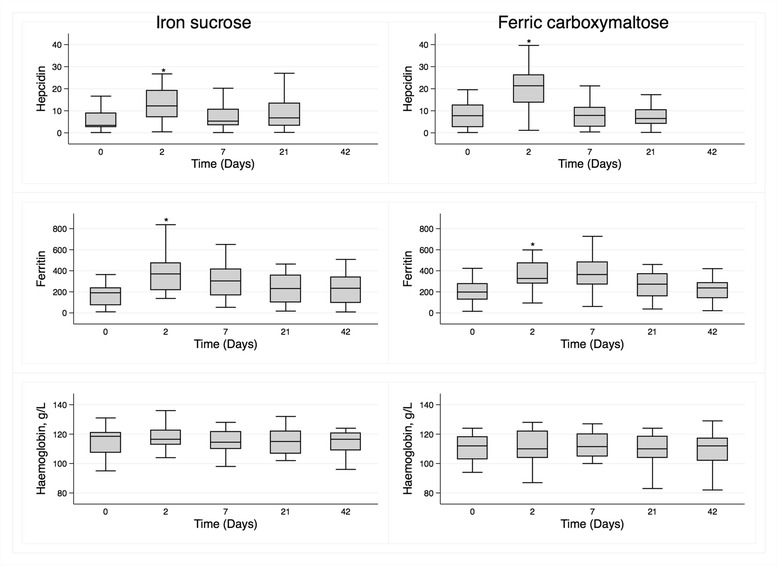
Median (interquartile range) levels over the course of the study of serum hepcidin (ng/mL), ferritin (μg/L) and haemoglobin (g/L) in participants treated with iron sucrose (*left panels*) and ferric carboxymaltose (*right panels*)

In the FCM Group, levels of iFGF23 (*p* = 0.046) and phosphate (*p* = 0.03) were lower at Day 2 whereas levels of cFGF23 (*p* = 0.036) were increased in pair-wise comparisons (Fig. 2, Table 3; Additional file 1: Figure S1). There was no significant change at Day 2 in iFGF23, cFGF23 or serum phosphate in the IS Group. Both serum hepcidin and ferritin increased markedly in both groups (*p* < 0.001, Fig. 3 and Table 3), with hepcidin returning to baseline more quickly than ferritin in the FCM Group (Additional file 1: Figure S2).

**Table 3:** Median (inter-quartile range) levels of iFGF23, cFGF23, hepcidin, ferritin and phosphate at Day 0 and Day 2, according to randomized treatment group

	Ferric carboxymaltose (*n* = 22)			Iron sucrose (*n* = 20)		
	Day 0	Day 2	P	Day 0	Day 2	*P*
iFGF23 (pg/mL)	843 (313–1922)	576 (356–1296)	0.046	381 (245–1526)	378 (235–1584)	0.77
cFGF23 (RU/mL)	704 (475–1204)	813 (267–1156)	0.036	710 (448–1548)	646 (307–1576)	0.23
Ferritin (μg/L)	198 (129–276)	327 (281–472)	<0.001	191 (77–237)	370 (218–475)	<0.001
Hepcidin (ng/mL)	7.8 (2.7–12.6)	21.4 (13.9–26.2)	<0.001	3.4 (2.7–8.8)	12.2 (7.3–19.2)	<0.001
Phosphate (mmol/L)	1.53 (1.14–1.71)	1.37 (1.05–1.67)	0.030	1.29 (1.11–1.65)	1.34 (1.08–1.54)	0.089

A linear mixed model with an interaction term for treatment and time demonstrated that the increase in serum hepcidin concentration from Day 0 to Day 2 was 4.2 ng/mL (95% CI: +0.4 to +8.0) greater in the FCM Group than the IS Group (p for interaction = 0.032). Addition of natural log-transformed CRP to this model to adjust for inflammation did not materially alter this finding (p for interaction 0.027). However, the changes in log-transformed iFGF23, cFGF23, ferritin, and serum phosphate, were not significantly different between treatment groups.

### All time points from day 0 to day 42

Considering all time points from Day 0 to Day 42 and adjusting for multiple comparisons, median serum hepcidin and ferritin levels differed significantly over time in both randomized groups, whereas iFGF23, cFGF23, and serum phosphate did not change (Additional file 2: Table S1).

### Exploratory analysis by reported daily urine volume

**Fig. 4:**
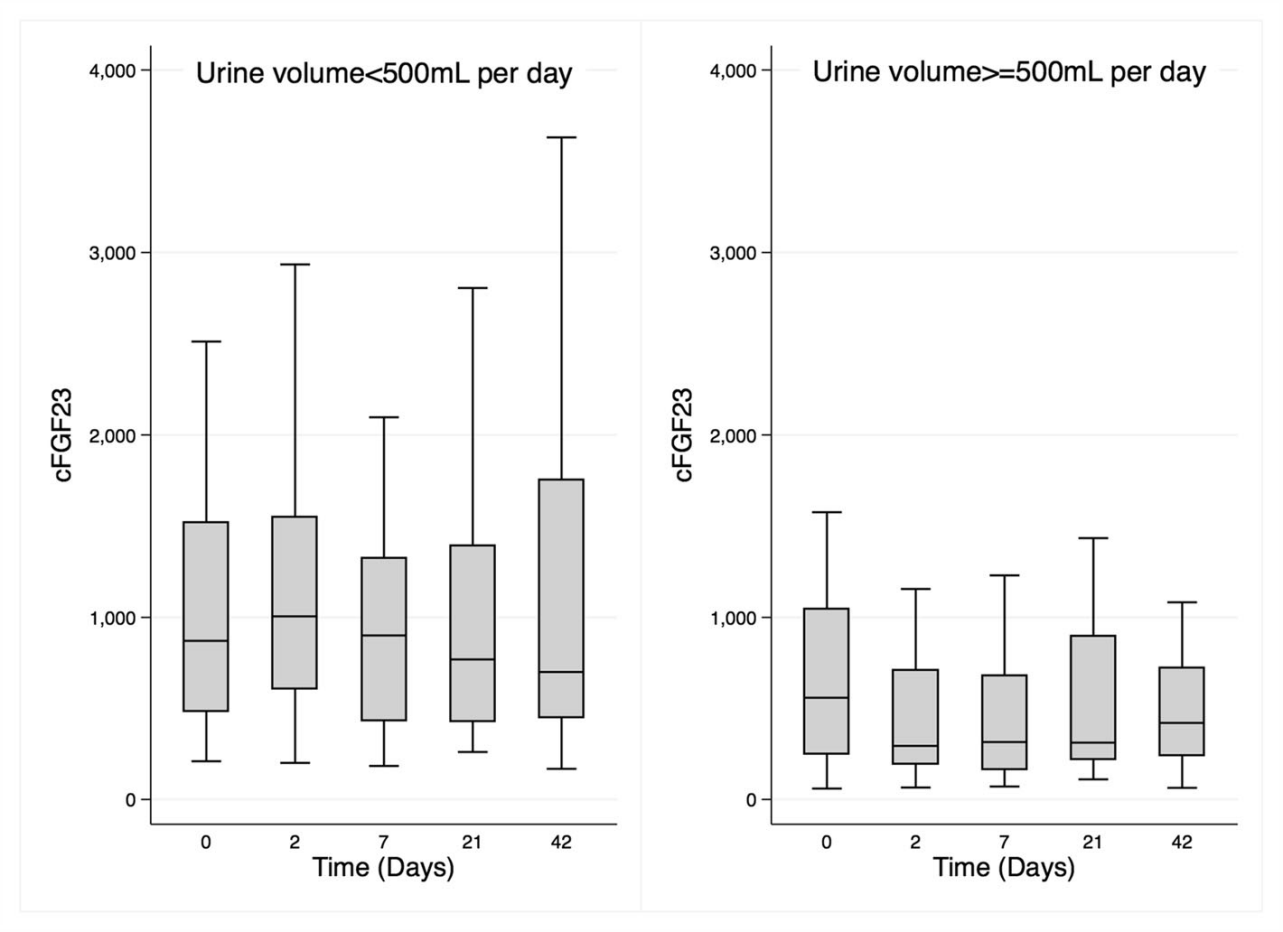
Median levels of cFGF23 over time in participants with low (<500 mL) daily urine output (*left panel*) and higher (≥500 mL) daily urine output (*right panel*)

Exploratory analysis was performed on the whole group to determine whether self-reported daily urine output ≥500 mL (*n* = 16) versus <500 mL (*n* = 26) influenced the levels of iFGF23 and cFGF23. A linear mixed model adjusted for the effect of randomized treatment that included all measures over 42 days demonstrated that cFGF23 was 0.80 log-units lower (95% CI: −1.33 to −0.26, *p* = 0.003) in participants who passed ≥500 mL/day compared to those passing <500 mL/day. Addition of an interaction term for urine volume and time demonstrated that cFGF23 between Day 0 and Day 2 was 0.41 log-units lower (95% CI: −0.70 to −0.13, p for interaction = 0.004) in participants with urine volume ≥500 mL/day compared to <500 mL/day (Fig. 4). Separate analyses by treatment group demonstrated a greater lowering of cFGF23 in participants in the IS group with higher versus lower urine output by 0.60 log-units (−1.05 to −0.14, p for interaction = 0.01). The corresponding log unit change for FCM for higher versus lower urine output was −0.22 (−0.56 to +0.12, p for interaction = 0.22). These differences were not demonstrated for iFGF23, hepcidin, ferritin or phosphate.

## Discussion

In this randomized controlled trial comparing two standard intravenous iron formulations in HD patients, both groups received sufficient iron to stimulate an increase in both serum hepcidin and ferritin. Notably, the changes in hepcidin were significantly greater in the FCM Group which, unlike the IS Group, also displayed a modest drop in iFGF23 and serum phosphate, and a rise in cFGF23. Participants with an arbitrarily defined higher urine output (≥500 mL) had both a lower serum baseline cFGF23 and a greater fall after iron infusion (both FCM and IS) compared to those with lower daily urine volumes (<500 mL).

The direction and degree of change in iFGF23 and cFGF23 in our study differ from previous prospective studies, which demonstrated an increase in iFGF23 following iron, and a fall in cFGF23 [13, 20] (Table 4). Important differences between these past studies and ours include baseline ferritin and transferrin saturation values, the level of kidney function and the iron infusion regimens. Iron deficiency anaemia itself is associated with higher levels of FGF23 in laboratory [5] and clinical studies [13], and so the degree of iron availability in participants may influence baseline levels of FGF23. Our participants were not iron deficient in the accepted sense but did require intravenous iron according to a standardised protocol. Furthermore, we administered a single 200 mg dose. Prior studies in HD patients administered either a much higher dose (mean 450 ± 131 mg) [14], or multiple smaller doses at each HD session over 3 weeks [15]. We did not do this because we wanted to see the effect of a single dose over time, rather than cumulative effects of multiple doses.

**Table 4:** Summary of findings compared to other prospective studies that administered intravenous iron and measured FGF23.

Study:	Current study		Hryszko, 2012 [14]	Takeda, 2011 [15]	Prats, 2013 [20]	Wolf, 2013 [13]		Schouten, 2009 [12]
Patient group	HD		HD	HD	CKD	Iron deficient women, no CKD		Medical outpatients, no CKD
Number of participants	20	22	12	27	47	25	30	8
Baseline ferritin (μg/L)	187 ± 130	211 ± 133	64.4 ± 32.7	31 ± 23	67.8 ± 61.7	4.4 ± 0.6	6.9 ± 1.7	
Iron formulation	IS	FCM	Iron dextran	Saccharated ferric oxide	FCM	FCM	Iron dextran	Iron polymaltose
Iron dose regimen:	1 dose of 200 mg	1 dose of 200 mg	1 dose individualised, mean = 450 mg	9 doses of 40 mg after dialysis (total = 360 mg)	1 dose (15 mg/kg to a maximum of 1000 mg, mean = 972 mg)	1 dose (15 mg/kg to a maximum of 1000 mg, mean = 918 mg)	1 dose (15 mg/kg to a maximum1,000 mg, mean = 911 mg)	1 dose (mean = 918 mg, range 500–1600 mg)
FGF23 measures post-infusion:	2, 7, 21, 42 days	2, 7, 21, 42 days	Weeks 1 & 3	Weeks 1, 3 & 5	Week 3 & 12	Days 1, 7, 14, 28 & 35	Days 1, 7, 14, 28 & 35	Weekly for up to 7 weeks
iFGF23	−	↓	↑	↑		↑^a^	−	↑↑
cFGF23	−	↑			↓	↓↓	↓↓	
Ferritin	↑↑	↑↑	↑	↑	↑	↑↑^a^	↑	
Hepcidin	↑	↑↑^a^				↑	↑↑^a^	
Phosphate	−	↓	−	−	↓	↓	−	↓

*CKD* chronic kidney disease, *iFGF23* intact fibroblast growth factor 23, *cFGF23* c-terminal fibroblast growth factor 23, *HD* haemodialysis patients, *FCM* ferric carboxymaltose, *IS* iron sucrose

Blank cells indicate that the analyte was not measured, ‘-’ indicates no change

^a^Significant between-group difference with time

Kidney dysfunction itself is known to affect the relative levels of iFGF23 and cFGF23. In CKD, the transcription and release of iFGF23 is thought to be partially uncoupled, with a reduced rate of cleavage to inactive N-terminal and C-terminal fragments [4]. C-terminal FGF23 fragments as a proportion of the total FGF23, determined by volumetric quantitation of Western Blot analysis at appropriate molecular weights of 14 kDa (cFGF23) and 32 kDa (iFGF23), decline as renal function falls: 21–56% with normal kidney function, 19% in pre-dialysis CKD patients, and 5% in HD patients [21]. The relatively low level of cFGF23 in HD patients, which was not measured in the previous studies in HD patients [14, 15], may explain why we did not demonstrate a fall in this marker, as others have in non-HD patients. Furthermore, residual kidney function in HD patients appears to affect FGF23 regulation and its ability to stimulate urinary phosphate excretion, possibly through an associated deficiency in Klotho. We did not assess phosphate excretion, and so could not determine whether changes in serum phosphate were explained by enhanced excretion (via sodium-phosphate co-transporters) or mediated by another mechanism such as intracellular uptake. It is also unclear precisely when the changes in phosphate occurred. However, the greater reduction in cFGF23 after iron infusion in participants with a higher urine output, a change akin to what was seen in the non-HD studies [13, 20], suggests that residual renal function in HD patients may affect the response to intravenous iron as well as modulate circulating FGF23 concentrations independent of serum phosphate. These data raise the question whether FGF23 is able to modulate fractional phosphate excretion in the face of reduced kidney function, and whether urine output is relevant to iron and phosphate homeostasis even in the end-stage kidney disease population. No studies have yet been performed on dialysis patients with significant residual renal function.

In this randomized controlled trial, we were able to confirm that responses to different iron formulations can vary, as demonstrated previously in iron-deficient women [13]. In our study, serum hepcidin increased markedly in both groups following iron infusion, but to a greater extent in the FCM Group, despite equivalent doses of elemental iron. Furthermore, at least at the limited time points studied, the rise in hepcidin appeared to occur before the increase in ferritin. No associations or correlations were identified between the hepcidin response and change in FGF23, and it is unclear what the different mediating effects on hepcidin might be other than the significant differences associated with respective formulations. Inherent structural differences in the carbohydrate shell of the respective iron formulations, resulting in different rates of release of labile iron into the serum [22] may at least be a partial explanation.

The clinical importance of differences in FGF23 stimulation between iron formulations can be considered immediate or long term. The immediate effect of FGF23 is lowering of serum phosphate which in our study was only modest, but has been demonstrated in other studies (Table 4). A potential longer term effect of concern is that higher levels of FGF23 are associated with both cardiovascular events and death in advanced CKD [23] and that FGF23 is associated with left ventricular hypertrophy (LVH) in human studies [24]. Experimental studies have identified that the LVH associated with elevated FGF23 serum levels and mediated by its specific cardiac receptor (Type 4) can be mitigated by blocking the receptor [25]. If an iron formulation has less effect on FGF23 production, it may potentially have fewer adverse cardiac effects.

Strengths of this study are that we compared iron formulations in a randomized controlled trial, that we assessed the inherent variability in iFGF23 before infusion, measured both iFGF23 and cFGF23, and examined them at an earlier time point (Day 2) than previously studied in HD patients. Although we collected samples at an earlier time point to measure FGF23 than previous studies in HD patients, more frequent collection either side of day 2 may better characterise the trajectory of change in FGF23. The within-subject variability in iFGF23 was a significant limitation as it reduced our ability to detect a change in iFGF23 with time and some reduction may have just been regression to the mean. This high within-subject variability has also been demonstrated in healthy volunteers although at a much lower level [21], and suggests that some of the changes demonstrated could be due to biological fluctuation. We tried to address inherent variability by taking the mean of 3 pre-infusion measurements, although results were not different when the analyses were repeated using a single baseline iFGF23 sample. To minimise between-subject variability, the baseline sample was collected before the second (“middle”) dialysis day of the week in all participants. In view of the variability in FGF23, the sample size was small and increasing the sample size may have helped to overcome this limitation. Another way to address this would have been to include a control group who received no iron but have FGF23 measured at the same time points. When we commenced the study, we were concerned about not giving iron to patients who met the criteria for receiving intravenous iron (based on international guidelines). However, given the demonstrated variability of FGF23 in this group of patients, this will be an important consideration for future studies.

## Conclusion

Infusion of 200 mg of intravenous FCM to non-iron-deficient, stable HD patients resulted in a significant reduction in iFGF23 and phosphate, and a rise in cFGF23. Little if any change was seen in the IS Group and no between-group changes were identified for these variables. Evidence of altered iron handling was established with marked increases in hepcidin and ferritin, with a significant between-group difference in hepcidin (and greater rise in the FCM Group). Residual urine output might also have had an effect on FGF23 concentrations in this end-stage kidney disease population. Future studies examining the effect of iron on the regulation of FGF23 transcription and proteolysis should take into account the greatly augmented serum concentrations and substantial variability of FGF23 in this population, and the different iron handling responses to different formulations.

## Additional files

Additional file 1: Figures S1 and S2.
**Figure S1**. The individual participant levels of iFGF23 (pg/mL), cFGF23 (RU/mL) and phosphate (mmol/L) over the course of the study for participants randomized to iron sucrose (left panels) and ferric carboxymaltose (right panels). **Figure S2**. The individual participant levels of serum hepcidin (ng/mL) ferritin (μg/L), and haemoglobin (g/L) over the course of the study for participants randomized to iron sucrose (left panels) or ferric carboxymaltose (right panels). (PDF 670 kb)
Additional file 2: Table S1. Median (IQR) levels of iFGF23, cFGF23, ferritin, hepcidin and phosphate at Day 0, 2, 7, 21 and Day 42, by randomized treatment group. (PDF 93 kb)

## Abbreviations

CKD: Chronic kidney disease
CV: Co-efficient of variation
ESA: Erythropoiesis stimulating agent
FCM: Ferric carboxymaltose
FGF23: Fibroblast growth factor 23
HD: Haemodialysis
HIF: Hypoxia inducible factor
IS: Iron sucrose
LVH: Left ventricular hypertrophy
