# Supplementary material for: Effects of intravenous iron on fibroblast growth factor 23 (FGF23) in haemodialysis patients: a randomized controlled trial
Source: BMC Nephrol. 2016 Nov 16;17:177. doi: 10.1186/s12882-016-0391-7 (PMC5112660; doi:10.1186/s12882-016-0391-7)

Supplementary Figure

Figure S1. The individual participant levels of iFGF23 (pg/mL), cFGF23 (RU/mL) and phosphate (mmol/L) over the course of the study for participants randomized to iron sucrose (left panels) and ferric carboxymaltose (right panels).

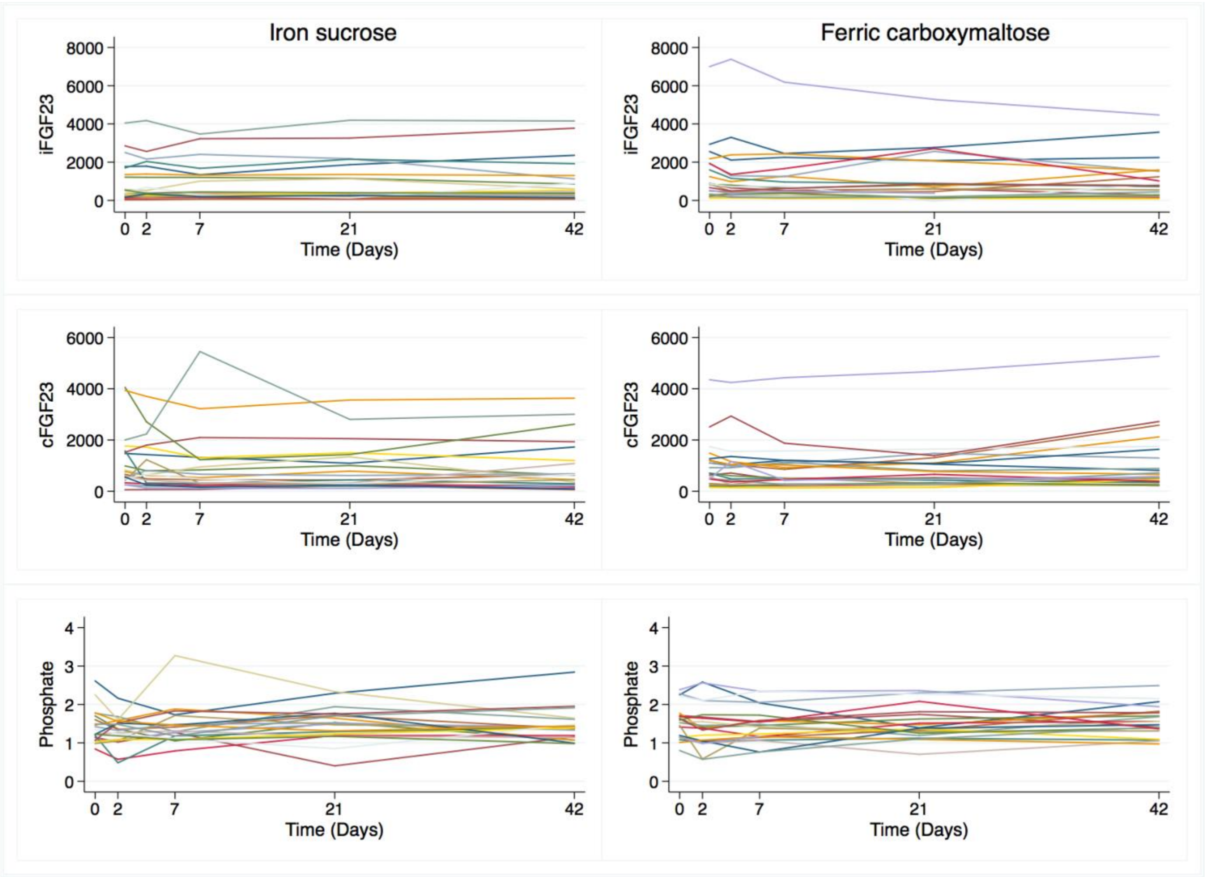

Figure S2. The individual participant levels of serum hepcidin (ng/mL) ferritin (µg/L), and haemoglobin (g/L) over the course of the study for participants randomized to iron sucrose (left panels) or ferric carboxymaltose (right panels).

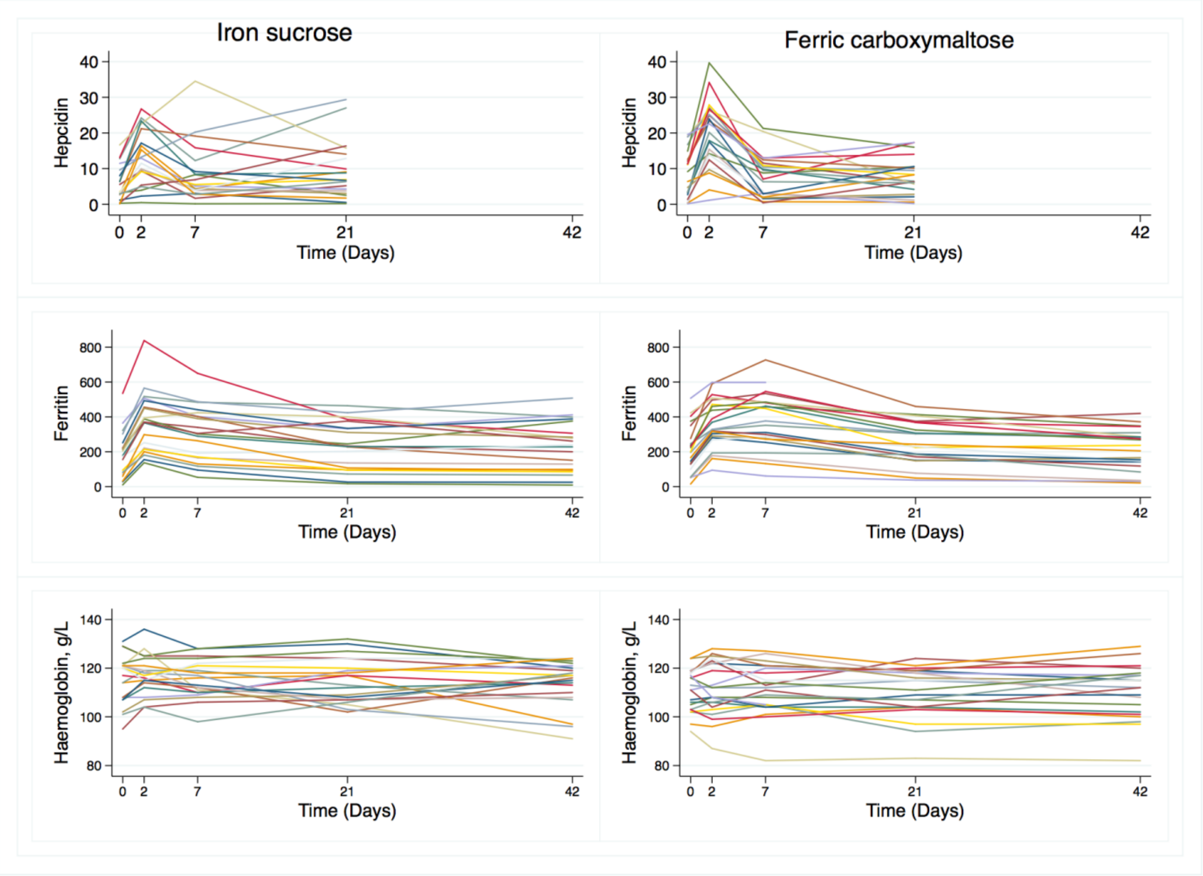

Supplement: Additional file 1: Figures S1 and S2. — Figure S1. The individual participant levels of iFGF23 (pg/mL), cFGF23 (RU/mL) and phosphate (mmol/L) over the course of the study for participants randomized to iron sucrose (left panels) and ferric carboxymaltose (right panels). Figure S2. The individual participant levels of serum hepcidin (ng/mL) ferritin (μg/L), and haemoglobin (g/L) over the course of the study for participants randomized to iron sucrose (left panels) or ferric carboxymaltose (right panels). (PDF 670 kb) [file 12882_2016_391_MOESM1_ESM.pdf]
