# Supplementary material for: Effects of intravenous iron on fibroblast growth factor 23 (FGF23) in haemodialysis patients: a randomized controlled trial
Source: BMC Nephrol. 2016 Nov 16;17:177. doi: 10.1186/s12882-016-0391-7 (PMC5112660; doi:10.1186/s12882-016-0391-7)
Supplement: Additional file 2: Table S1. — Median (IQR) levels of iFGF23, cFGF23, ferritin, hepcidin and phosphate at Day 0, 2, 7, 21 and Day 42, by randomized treatment group. (PDF 93 kb) [file 12882_2016_391_MOESM2_ESM.pdf]

Supplementary Table

Table S1. Median (IQR) levels of iFGF23, cFGF23, ratio of cFGF23 to iFGF23, hepcidin and phosphate at Day 0, 2, 7, 21 and Day 42, by randomized treatment group. P values for comparison assess whether median values differ across the whole time period and are adjusted for multiple comparisons. Unless indicated, P values for pair-wise comparison of specific time points, adjusted for multiple comparisons, were not significant.

	Iron sucrose						FCM					
	**Day 0**	**Day 2**	**Day 7**	**Day 21**	**Day 42**	**P**	**Day 0**	**Day 2**	**Day 7**	**Day 21**	**Day 42**	**P**
**iFGF23**	381 (245-1,526)	378 (235-1,584)	392 (228-1,330)	381 (178-1,614)	481 (165-1,214)	0.99	843 (313-1,922)	576 (356-1,296)	570 (253-1,264)	610 (164-2,064)	765 (275-1,519)	0.96
**cFGF23**	710 (448-1,548)	646 (307-1,576)	505 (267-1,269)	530 (275-1,388)	637 (279-1,460)	0.86	704 (475-1,204)	813 (267-1,156)	509 (294-1,134)	524 (300-1,078)	629 (372-1,639)	0.93
**Ferritin**	191 (77-237)	370 (218-475)[1]	304 (168-413)	232 (102-355)	234 (97-341)	0.005	198 (129-276)	327 (281-472)[1]	365 (272-484)[1]	273 (161-369)	237 (142-285)[4. 5]	<0.001
**Hepcidin**	3.4 (2.7-8.8)	12.2 (7.3-19.2)[1]	5.3 (3.5-10.7)[2]	6.8 (3.4-13.5)[3]	-	0.004	7.8 (2.7-12.6)	21.4 (13.9-26.2)[1]	7.9 (2.9-11.6)[4]	6.5 (4.2-10.5)[4]	-	0.004
**Phosphate**	1.29 (1.11-1.65)	1.34 (1.08-1.54)	1.3 (1.15-1.60)	1.51 (1.21-1.73)	1.39 (1.15-1.55)	0.65	1.53 (1.14-1.71)	1.37 (1.05-1.67)	1.43 (1.16-1.58)	1.37 (1.22-1.68)	1.56 (1.36-1.79)	0.64

[1]P<0.001 v Day 0; [2]P=0.019 v Day 2; [3]P=0.042 v Day 2; [4]P<0.001 v Day 2; [5]P<0.001 v Day
